# Associations of inflammatory cytokines with palmoplantar pustulosis: a bidirectional Mendelian randomization study

**DOI:** 10.3389/fmed.2024.1387210

**Published:** 2024-05-31

**Authors:** Chengling Liu, Xingchen Liu, Haiming Xin, Xin Li

**Affiliations:** ^1^Center of Burns and Plastic Surgery and Dermatology, The 924th Hospital of Joint Logistics Support Force of the PLA, Guilin, China; ^2^Department of Pathology, Changhai Hospital, Naval Medical University, Shanghai, China

**Keywords:** palmoplantar pustulosis, cytokine, Mendelian randomization, CXCL6, genome-wide association study

## Abstract

**Background:**

Variations in circulatory cytokine levels have been observed during the onset and course of palmoplantar pustulosis (PPP); however, whether these changes are due to etiological or secondary factors is unclear. To clarify the causal relationship, we conducted a summarized-level bidirectional Mendelian randomization (MR) analysis in this study.

**Methods:**

A FinnGen biobank genome-wide association study (GWAS) of 212,766 individuals (524 PPP patients and 212,242 controls) provided summary data for PPP, whereas genetic instrumental variables (IVs) linked to circulation cytokine levels were gathered from a GWAS of 14,824 European individuals. The inverse-variance weighted (IVW), weighted median (WME), simple mode, and MR-Egger methods were used to ascertain the changes in PPP pathogenic cytokine taxa. Sensitivity analysis, which included horizontal pleiotropy analysis, was then conducted. The reliability of the results was assessed using the leave-one-out approach and the MR Steiger test, which evaluated the strength of a causal relationship. To evaluate the reverse causality between PPP and circulating cytokine levels, a reverse MR analysis was carried out.

**Results:**

Our study demonstrated positive associations between C-X-C motif chemokine 6 (CXCL6) and PPP (odds ratio, OR 1.257, 95%CI: 1.001–1.570, *p =* 0.043). C-C motif chemokine 19 (CCL19) and interleukin-6 (IL-6) were suggested to be protectively associated with the development of PPP (OR: 0.698,95% CI: 0.516–0.944, *p =* 0.020; OR: 0.656, 95%CI:0.437–0.985, *p =* 0.042). The results were steady after sensitivity and heterogeneity analyses.

**Conclusion:**

At the genetic prediction level, we identified causally connected inflammation-related variables that contributed to the onset and development of PPP. The therapeutic options for some refractory PPP have expanded due to tailored cytokine therapy, generating fresh concepts for PPP diagnostics and mechanism investigation.

## 1 Introduction

Palmoplantar pustulosis (PPP), also known as pustulosis palmaris, is a recurring and persistent inflamed skin condition that presents with clinical features of multiple sterile pustules, scales, and erythema on the palms and soles (1, 2). According to research, the prevalence of PPP varies from 0.050% in Sweden, 0.051% in Korea, 0.091% in Germany, and 0.12% in Japan (3). PPP is more frequent in women; the frequency in studies conducted in Sweden and Japan ranged from 94 to 65.3% (4–6). More detrimental effects on quality of life are caused by this severe and resistant condition than other inflammatory skin lesions elsewhere on the body (7, 8). Numerous research investigations have proposed that environmental factors, medication consumption, metal allergies, stress, smoking cigarettes, and localized infections may have a role in the development or exacerbation of PPP (9–11). Meanwhile, environmental variables and the innate and adaptive immune systems have an interactive impact on PPP. Intraepidermal sweat duct expression of IL-17 has been observed, showing that the eccrine sweat gland is an immune-competent structure and an active part of the epidermal barrier (12). Increased Langerhans cells adjacent to eccrine sweat ducts reveal the presence of an antigen-driven mechanism that breaks down the acrosyringium by allowing mast cells, neutrophils, eosinophils, and lymphocytes to infiltrate. Compared to healthy people, patients with PPP had higher blood levels of TNF-α, IL-22, IL-36, and IFN-γ, indicating that their inflammatory pattern is comparable to psoriasis (13–16). In terms of PPP therapy, although biologics such as TNF-α inhibitors, IL-17, IL-23, IL-36, and IL-8 monoclonal antibodies have had some success, the outcomes have not been very encouraging yet (17–21). The authors examine the possibility that additional inflammatory variables contribute to the pathophysiology of PPP. Thus, by establishing a solid basis, future research focused on the causative link between circulating inflammatory variables and PPP might identify potential pathogenetic elements and determine the course of the subsequent targeted therapy.

In the past 10 years, genome-wide association studies (GWASs) have transformed the field of complex disease genetics by testing millions of genetic variations in several individual genomes to find genotype–phenotype relationships (22). Mendelian randomization (MR) research is utilized to identify the causal relationships between several genetically predicted exposures and complicated disorders (23). Genetic variations are used in MR as instrumental variables (IVs) to assess the causal connection between exposure and outcome (24). One benefit of this strategy is that it reduces bias and reverse causality caused by confounding variables. Researchers can evaluate the relationships between IVs and exposure and results across two different population samples by conducting a two-sample MR analysis, which increases the test’s usefulness and application (25). To explore the associations between 91 inflammatory cytokines and PPP, we first extracted valid genetic variants from the published GWAS summary data. We then investigated the direction of causation by reversing the exposures and outcomes.

## 2 Methods

### 2.1 Ethics statement and overall study design

**Figure 1:**
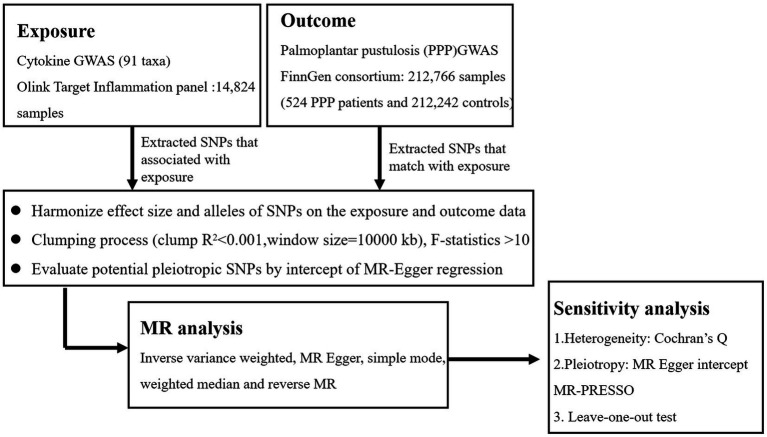
A flowchart of the Mendelian randomization study.

Previously released GWAS summary statistic was used in this MR analysis. Each institution’s review board and ethics committee approved the written informed consent for each participant in each study. Additional informed consent or further ethical approval was not necessary. The STROBE-MR checklist was rigorously followed when conducting this study (26). The flowchart in Figure 1 illustrates the entire procedure investigated. The three fundamental presumptions of the MR analysis are exclusion restriction, independence, and relevance. In the risk factor–outcome association (independence), it is assumed that the chosen genetic variants are associated with the risk factor (relevance) but not with any confounders and that they are not associated with the outcome through any other pathway than the risk factor of interest (exclusion restriction) (27). The inverse-variance weighted (IVW), MR-Egger regression, weighted median (WME), and simple mode are examples of two-sample MR methods that we utilized to look for any bidirectional causal relationships between cytokines and PPP. The pleiotropy test, heterogeneity test, leave-one-out sensitivity analysis, MR Steiger test, and MR-PRESSO were then carried out. Finally, the reverse MR analysis was carried out to evaluate the reverse causal connection between PPP and circulating cytokine levels.

### 2.2 Data sources

Table 1 presents the GWAS summary data that were utilized in the investigation. The individuals from the data sources were of European ancestry.

**Table 1:** Summary of genome-wide association study (GWAS) datasets.

Trait	Year	Population	Sources	Sample sizes	Cases	Control
*Exposure*						
Cytokine	2023	European	Finnish cohorts	14,824	–	–
*Outcome*						
PPP	2021	European	FinnGen biobank	212,242	524	212,766

#### 2.2.1 Cytokines

We obtained summary-level genome-wide association data for 91 circulating cytokines and PPP. Applying the Olink Target Inflammation panel, genome-wide pQTL mapping for 91 plasma proteins was carried out in 11 cohorts, which included a combined total of 14,824 individuals of European ancestry (28). Significant relationships identified by the discovery meta-analysis were evaluated for replication in an independent cohort (ARISTOTLE) of 1,585 people with Olink plasma proteome data to validate the pQTL results (29). After adjusting for age, sex, and body mass index, a linear regression analysis was performed between cytokine levels and SNPs to determine the single-variant link.

#### 2.2.2 Palmoplantar pustulosis

Summary statistics were derived from publicly accessible GWAS analyses of PPP in people of European ancestry. Over 16 million genetic variations were examined in the research, focusing on PPP patients (524) and controls (212,242).^1^ Written informed consent was provided by each participant for the study, which was authorized by the local institutional review boards.

### 2.3 Selection of genetic instrumental variables

In the initial stages, we used a *p*-value of <5 × 10^−8^ as the genome-wide significance level to identify significantly associated SNPs connected to inflammatory cytokines and PPP. However, a higher cutoff (*p* < 5 × 10^−6^) was used because there were only a few SNPs for several inflammatory cytokines when considered as exposure (30, 31). The following step we took to prevent linkage disequilibrium was SNP clumping (kb = 10,000, *r*^2^ = 0.001). Palindromic SNPs were excluded from the GWAS of systemic inflammatory regulators, as it was unclear whether they would align in the same orientation for exposure and outcome. Chromosome, effect allele (EA), other allele (OA), effect allele frequency (EAF), effect sizes (β), standard error (SE), and *p*-values were among the pertinent data that we retrieved. Finally, to ascertain if the identified IVs were substantially correlated with exposure, we computed the *F*-statistic (*F* = *R*^2^(n-k-1)/k(1-*R*^2^)), where *n* is the sample size, *k* is the number of included IVs, and *R*^2^ is the exposure variance explained by the chosen SNPs parameters and the explained variance (*R*^2^) (32). SNPs with *F*-statistic parameters less than 10 are often regarded as poor instruments (33).

### 2.4 Statistical analysis

The primary study utilized the IVW approach to objectively assess the causal association between cytokines and PPP (34). Mostly, exponential odds ratios (ORs) and their associated confidence intervals (CIs) were used to assess the causal impact sizes. The threshold for statistical significance was set at a *p*-value of <0.05. The MR-Egger, WME, and simple mode methods were also used to measure the causal effects under various circumstances. The WME technique can yield a valid estimate by merging data from several genetic variants into a single causal estimate, provided that at least half of the weight is generated from reliable IVs (33). If genetic mutations show directional pleiotropy, the MR-Egger method may be used to quantify the causal influence (35). The MR-Egger regression intercept and MR pleiotropy residual sum and outlier (MR-PRESSO) were calculated to assess horizontal pleiotropy. Given that the *p*-value is more than 0.05, it is unlikely that pleiotropy would affect the causative analysis (36). To find heterogeneity across instrumental factors, Cochran’s *Q*-test was created using the IVW estimating approach (37). We also applied the MR pleiotropy residual sum technique to assess horizontal pleiotropy and eliminate any outliers. The leave-one-out method was used to determine the extent to which a single SNP contributed to the causal connection. This strategy entailed systematically removing one SNP and using the remaining SNPs as IVs for two-sample multiple regression analysis. An extensive evaluation of the relationship between exposure and results was conducted using the MR Steiger directionality test. The MR Steiger method states that sufficient genetic variation should account for more variance during exposures than during outcomes (38, 39). This method helps to identify any reciprocal effects and ensures that the genetic instruments are appropriate for a valid MR investigation. Finally, to investigate how PPP affects the identified cytokines, we performed a reverse magnetic resonance analysis. IVs were created using PPP-related SNPs.

We believed that there was a substantial causal relationship between systemic cytokines and PPP if the following criteria were met: The IVW method showed a significant difference (*p <* 0.05), the MR Steiger directionality tests verified TRUE, the four approaches produced consistent estimates, and the findings of Cochran’s Q-test, MR-Egger, and MR-PRESSO were not significant (*p* > 0.05) (40, 41). All MR studies were performed using the “TwoSampleMR” package (version 4.2.2) for the R program.

## 3 Results

### 3.1 Selection of instrumental variables

Following LD clumping and screening at a low threshold (*p <* 5 × 10^−6^), SNPs of cytokines were chosen. The resulting SNPs for the cytokine trait are presented in detail in Supplementary Table S1. Given that all of the F statistics for the IVs were more than 10, there was no minor instrument bias.

### 3.2 Causal impact of cytokine on PPP

**Figure 2:**
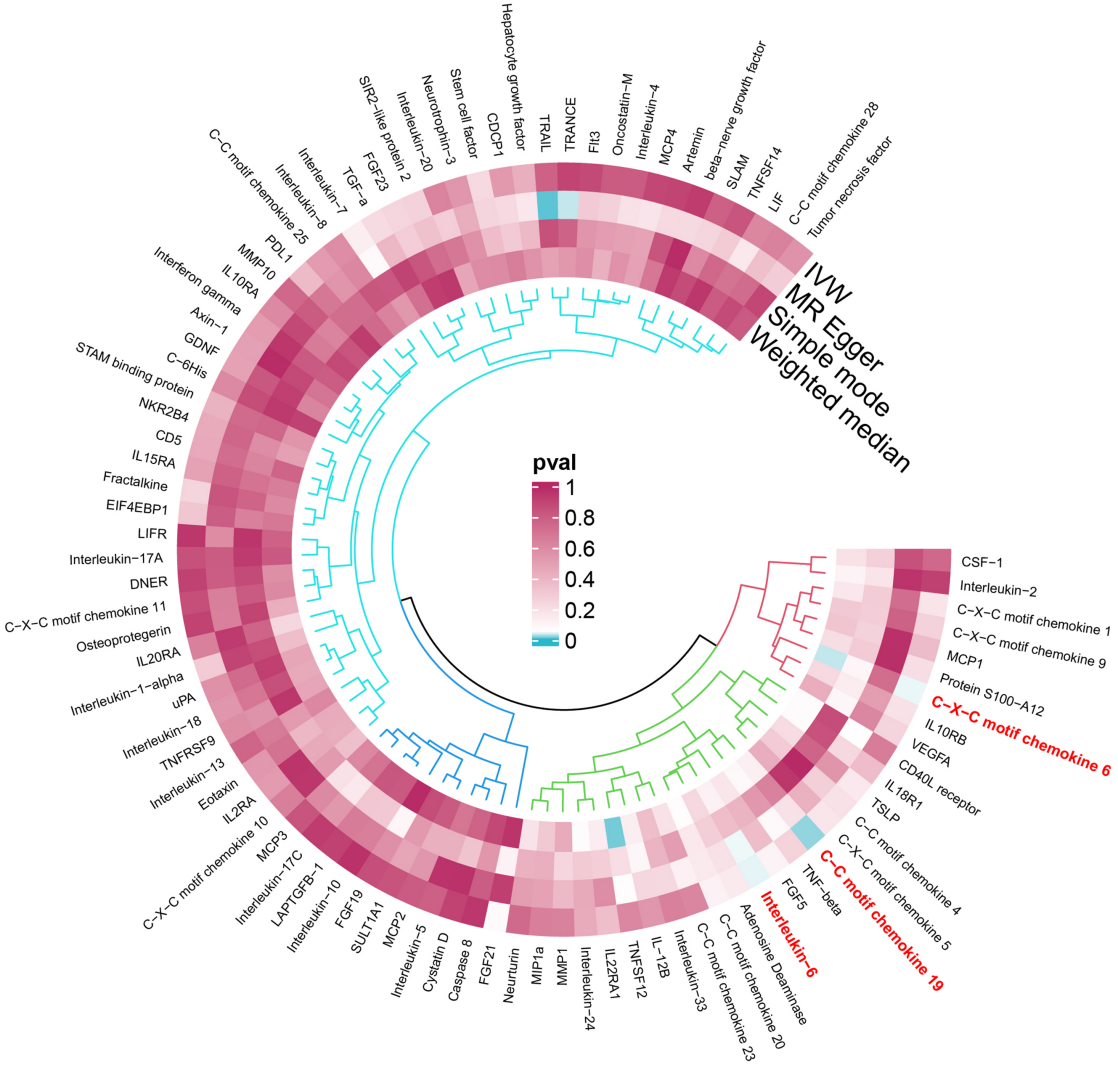
An overview of the causal role of cytokines in PPP. The cyan process color indicates statistical significance (*p* < 0.05). IVW inverse-variance weighted method.

An overview of the causal effect of 91 cytokines taxa on vitiligo is shown in Figure 2. Of all the cytokines, three were selected for further MR analyses. Furthermore, 13 independent SNPs were associated with C-C motif chemokine 19 (CCL19), 11 independent SNPs were associated with C-X-C motif chemokine 6 (CXCL6), and 8 independent SNPs were associated with interleukin-6 (IL-6). SNP detailed messages (SD, *R*^2^, *F*) of significant genera in MR analyses are shown in Table 2.

**Table 2:** SNP message of significant cytokines.

Cytokine	SNP	SD	*R*^2^	*F*
CCL19	rs7595241	1.553921491	0.001564131	22.38017751
	rs10496135	4.590045019	0.001632575	23.36111111
	rs13010492	1.424996274	0.003165857	42.6207284
	rs113539352	9.861090648	0.002064683	23.66263216
	rs62292952	2.245140976	0.010802168	160.8098174
	rs79086127	5.371235891	0.001520667	21.69489383
	rs4554017	1.458295553	0.001667774	23.86562752
	rs3792790	1.434389069	0.001473634	21.08340278
	rs6870560	2.051245115	0.001767166	26.0764329
	rs9469127	3.362442289	0.020664835	310.8792764
	rs28635588	1.973948713	0.008022714	74.24415591
	rs1415763	1.905529897	0.00157592	23.24836707
	rs10242459	1.86899161	0.001519284	22.40854276
CXCL6	rs12075	1.396243979	0.003404361	50.34827977
	rs4951518	4.862918082	0.001951244	22.13321586
	rs184103539	3.977466582	0.001621872	20.85704661
	rs16850073	1.410877401	0.110721007	1779.695777
	rs116381873	6.141951008	0.001844414	24.71831211
	rs191180456	7.61475826	0.002835109	39.25563938
	rs35096759	5.105463137	0.001703739	24.39480829
	rs9371716	2.150196707	0.001823694	22.67338721
	rs182381042	9.087198102	0.001774435	22.98956455
	rs4877818	1.439362109	0.001882249	21.75456672
	rs574432935	7.822355267	0.001921552	24.59701293
IL-6	rs2228145	1.444857993	0.01340393	200.2581739
	rs17486819	2.561630377	0.001446793	21.35228319
	rs248536	1.709732304	0.001538088	22.01775148
	rs550717566	1.56620348	0.001826921	26.15814929
	rs10234370	1.518433584	0.00146716	21.00093
	rs187416244	5.6913493	0.001552385	22.89285828
	rs1346147	1.614082944	0.001664395	23.82886145
	rs10977774	7.778257589	0.001927579	23.77431873

SD, standard deviation; SNP, single-nucleotide polymorphism.

**Figure 3:**
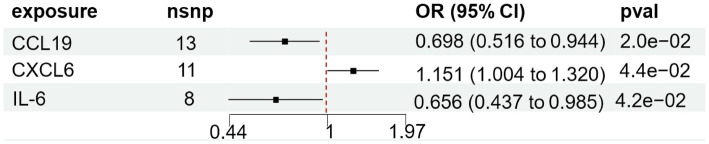
Forest plot of cytokines associated with PPP identified by the inverse-variance weighted method. SNP single-nucleotide polymorphism, OR odds ratio, CI confidence interval.

We found that CXCL6 (OR 1.257, 95%CI 1.01–1.57, *p =* 0.044) had an increased risk of developing PPP, while CCL19 (OR 0.698, 95%CI 0.52–0.94, *p =* 0.020) and IL-6 (OR 0.656, 95%CI 0.44–0.99, *p =* 0.042) had a protective role in PPP (Figure 3). For all outcomes, the MR Steiger directionality tests revealed a similar pattern from cytokine to PPP (Table 3).

**Table 3:** Significant MR analysis results.

Cytokine taxa	MR method	No. SNP	OR	95%CI	*p*-value	*p* for MR-PRESSO global test
CCL19	IVW	13	0.70	0.52–0.94	0.02	
	Weighted median		0.72	0.47–1.09	0.1236	
	MR-Egger		0.68	0.39–1.17	0.19	
	Simple mode		0.89	0.52–2.43	0.63	
	MR-PRESSO					0.31
CXCL6	IVW	11	1.26	1.01	1.57	
	Weighted median		1.20	1.02–1.42	0.03	
	MR-Egger		1.06	0.78	1.42	
	Simple mode		1.77	0.97–3.22	0.09	
	MR-PRESSO					0.14
IL-6	IVW	8	0.66	0.44–0.98	0.04	
	Weighted median		0.68	0.43–1.10	0.11	
	MR-Egger		0.38	0.18–0.81	0.04	
	Simple mode		0.71	0.36–1.41	0.36	
	MR-PRESSO					0.08

OR, odds ratio; CI, confidence interval; SNP, single-nucleotide polymorphism; MR, Mendelian randomization; MR-PRESSO, MR pleiotropy residual sum and outlier.

### 3.3 Sensitivity analysis

**Figure 4:**
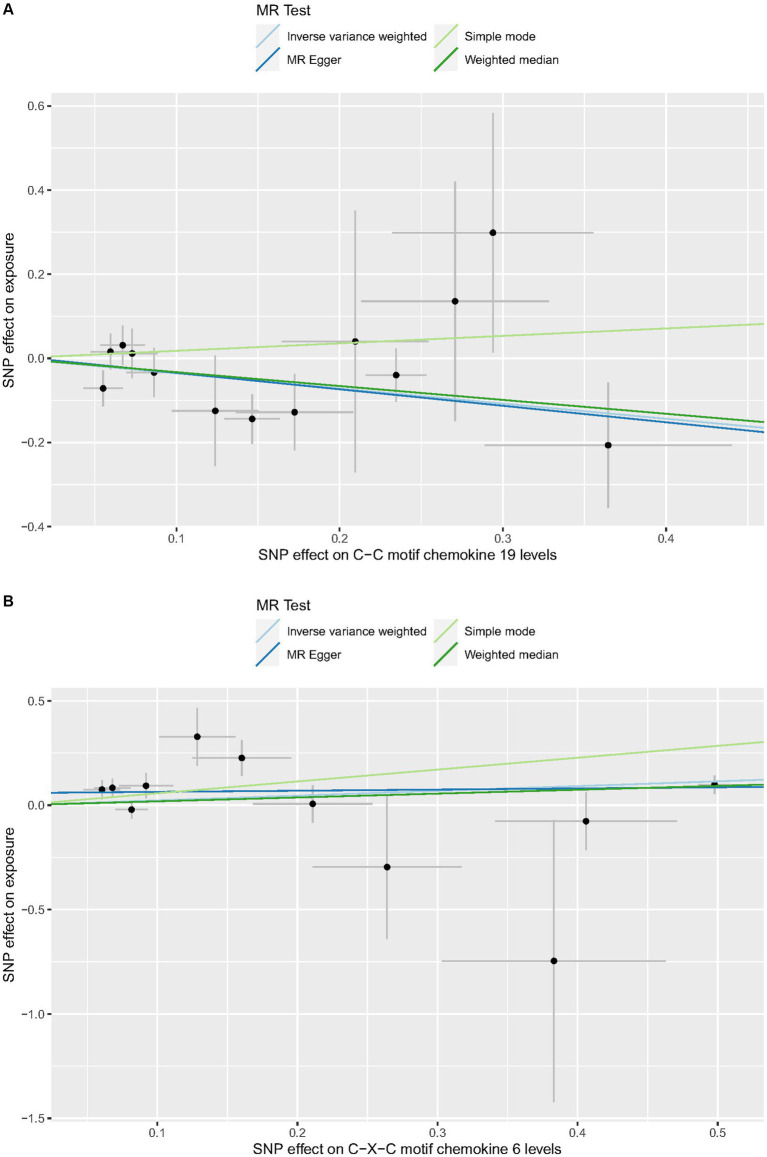

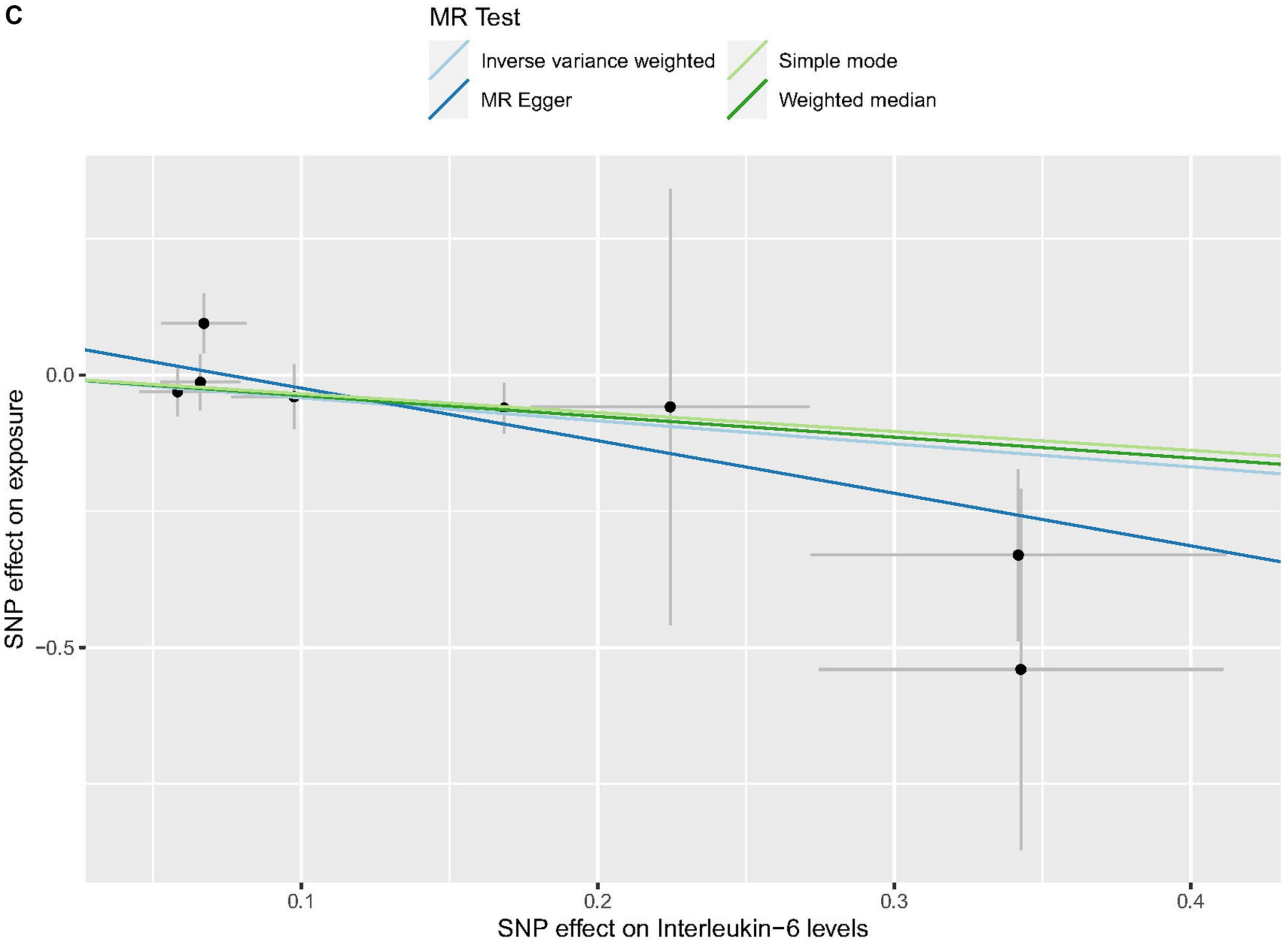
Scatter plots of cytokines associated with the risk of PPP. [**(A)** for CCL19, **(B)** for CXCL6, and **(C)** for IL-6].

**Figure 5:**
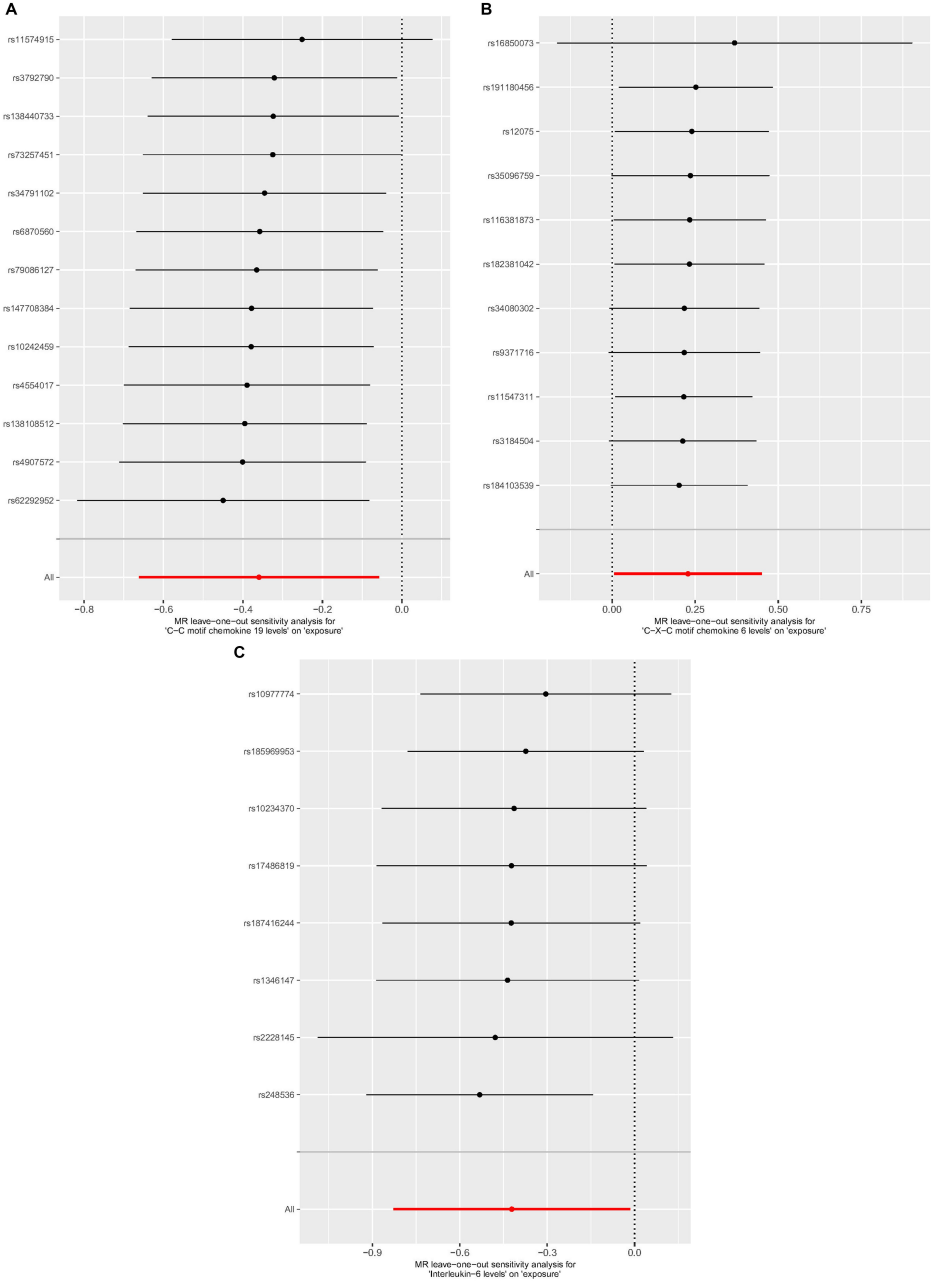
Leave-one-out analysis of cytokines associated with the risk of PPP. [**(A)** for CCL19, **(B)** for CXCL6, and **(C)** for IL-6].

**Figure 6:**
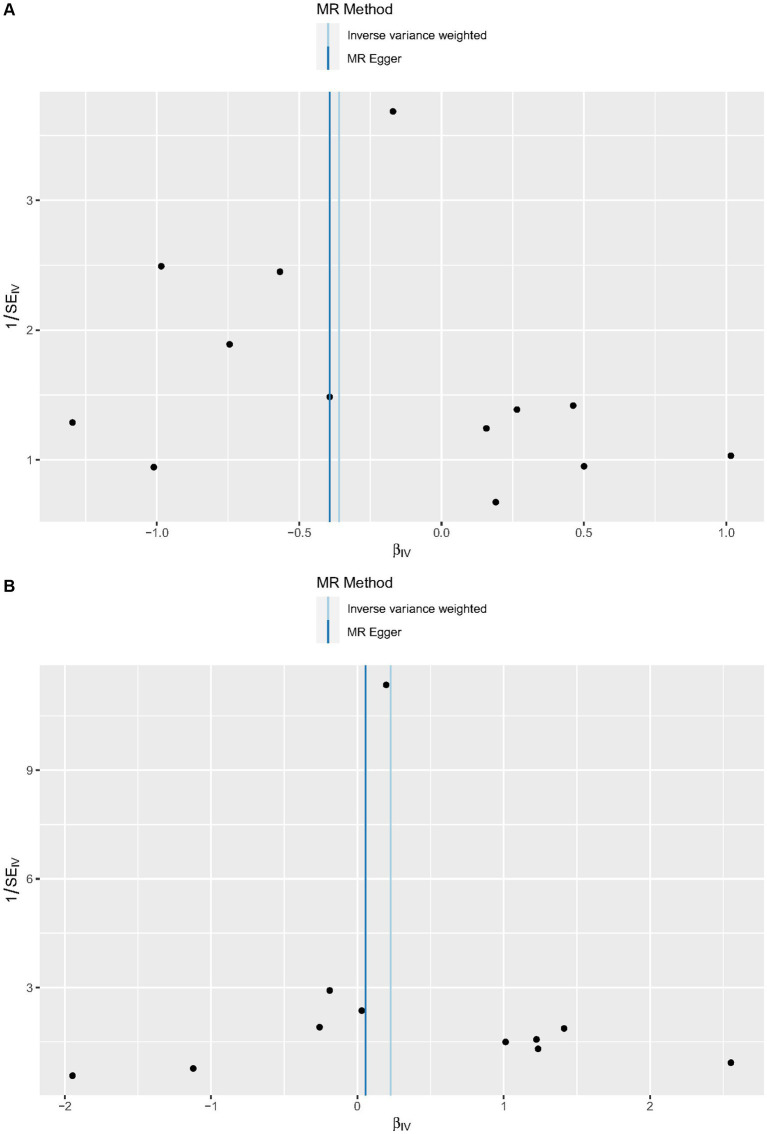

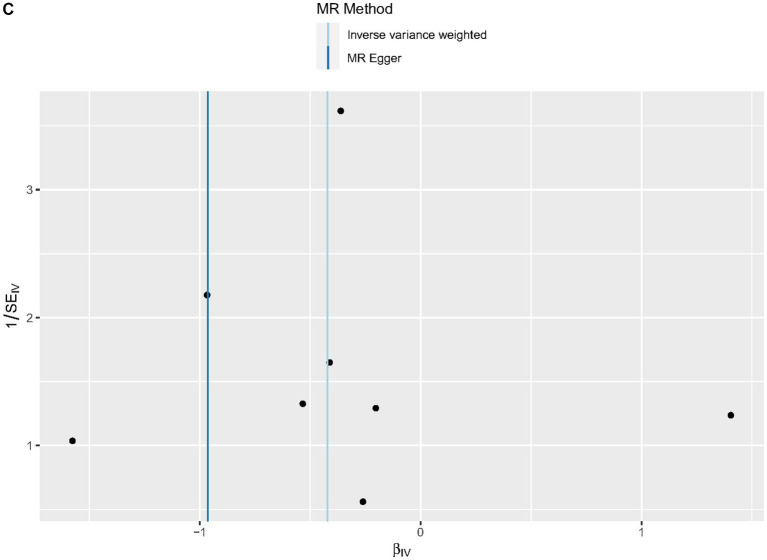
Funnel plots of cytokines associated with the risk of PPP. [**(A)** for CCL19, **(B)** for CXCL6, and **(C)** for IL-6].

Cochrane’s *Q*-test revealed no variability within the cytokine IVs (Table 4). According to the MR-Egger regression intercepts and MR-PRESSO, there were no horizontal pleiotropy or outlier values (*p* > 0.05). The scatter plots demonstrated that, while CCL19 and IL-6 might help prevent PPP, CXCL6 might be detrimental to that ability. The MR analysis techniques with weights depicted in the scatter plots include the IVW method, MR-Egger, WME, and simple mode. The lines that sloped upward from left to right were determined to be positive indicators of the link between the cytokines and PPP, whereas the lines that slid downhill from left to right were found to be protective cytokines (Figure 4). There were no potential outliers in the “leave-one-out” analysis of the cytokine IVs for PPP (Figure 5), indicating that the discovered causal relationship was not affected by a single IV. Furthermore, the funnel plots showed no appreciable horizontal pleiotropy for any given result (Figure 6). The reverse MR analysis revealed that PPP had no causative relationship between CCL19, CXCL6, and IL-6 (Table 5).

**Table 4:** Sensitivity analysis of cytokines associated with vitiligo.

Exposure	SNPs	MR-Egger intercept		Cochrane’s Q IVW		Cochrane’s *Q* Egger		Correct causal direction
		Intercept value	*p-*value	*Q*-value	*p-*value	*Q*-value	*p*-value	
CCL19	13	0.005	0.892	10.9	0.54	10.9	0.46	True
CXCL6	11	0.05	0.15	20.4	0.33	15.9	0.07	True
IL-6	8	0.07	0.15	8.1	0.32	5.4	0.50	True

SNPs, single-nucleotide polymorphism; IVW, inverse-variance weighted; MR. Mendelian randomization.

**Table 5:** Reverse causal association between PPP and cytokines.

Cytokines (outcome)	MR method	No. SNP	OR	95%CI	*p*-value
CCL19	IVW	9	0.98	0.82–1.16	0.780
	Weighted median		0.97	0.70–1.36	0.884
	MR-Egger		0.91	0.59–1.41	0.694
CXCL6	IVW	9	1.01	0.76–1.32	0.989
	Weighted median		1.20	0.93–1.52	0.16
	MR-Egger		1.22	0.60–2.48	0.60
IL-6	IVW	35	0.96	0.92–1.01	0.113
	Weighted median		0.95	0.84–1.07	0.374
	MR-Egger		0.93	0.84–1.03	0.187

SNPs, single-nucleotide polymorphism; IVW, inverse-variance weighted; MR. Mendelian randomization.

## 4 Discussion

Research has revealed a robust association between circulating cytokines and PPP; however, the underlying mechanisms behind this association remain unclear. Significant correlations between circulatory cytokines and PPP have been demonstrated in research. We used a two-sample MR to assess the causal association between circulating cytokines and PPP in our investigation, which used large-scale GWAS for 91 circulating cytokines and PPP. In our study, CCL19 and IL-6 (OR:0.698, 95% CI: 0.516–0.944, *p =* 0.020; OR: 0.656, 95%C1: 0.437–0.985, *p =* 0.042) were shown to have substantial beneficial associations with PPP, whereas CXCL6 (OR 1.257, 95%CI: 1.001–1.570, *p =* 0.043) was found to have significant detrimental connections. No reverse causal association between inflammatory variables and PPP was discovered in the reverse MR, and the results were robust and dependable according to heterogeneity and horizontal pleiotropy tests.

PPP is characterized by sterile pustules on an erythematous scaly substrate (42). There is a lack of understanding regarding confirmed predicted risk factors, and its pathophysiology is still not entirely understood (43). Regarding gene mutation, research indicates that fusion of the IL-36RN gene (which encodes the interleukin IL-36 receptor antagonist) results in increased expression of the inflammatory cytokine IL-36, potentially exacerbating the onset of PPP (44). Moreover, subsequent research has also revealed that GPP patients carried AP1S3 and CARD14 alleles, which could enhance the production of IL-36 in several ways (45, 46). For the inflammatory cytokines, PPP has a pathophysiological mechanism involving innate and acquired immunity. Blisters and pustules result from sweat gland inflammation within the epidermis (47). Eccrine sweat glands express more IL-17, indicating a potential role for this location in the pathophysiology (48). Moreover, serum levels of TNF-α, IL-17, IL-22, and IFN-γ have been elevated in PPP patients (49–52). Research showed that keratinocytes at PPP lesions expressed IL-36R and that these cells could secrete IL-17A in a positive feedback loop to regulate IL-36 secretion. Additionally, keratinocytes at PPP lesions could produce inflammatory cytokines and chemokines through IL-36 self-activation. Finally, IL-36 could stimulate dendritic cells (DC) to produce many IL-23 differentiated T cells, eventually becoming Th17 cells. As a result, in PPP, the IL-36, IL-23, and IL-17 axis’ reciprocal stimulation increases the inflammatory response and is crucial to the development of PPP (53). Therefore, the common biologic that is presently approved for the treatment of PPP in Japan is guselkumab (50), an anti-IL-23 antibody that has shown promising clinical results. Nevertheless, not every PPP patient responded well to guselkumab. In patients who did not respond well to guselkumab therapy, several researchers modified the use of small molecule medicines, such as JAK inhibitors, and obtained effective results (54). This suggests that PPP may be influenced by inflammatory variables other than IL-23 and IL-36. Thus, research on these inflammatory variables implicated in PPP pathophysiology can open up new therapeutic avenues and provide PPP patients who have not responded to traditional therapy with hope for a new course of treatment.

A family of ubiquitous, chemotactic, and inducible small molecule peptides known as chemokines are widely involved in acute and chronic inflammation (55). CXCL6 is a type I membrane protein containing a non-ELR motif-containing CXC chemokine domain that attracts inflammatory cells (such as neutrophile granulocytes) to the site of inflammation by binding to the receptors CXCR1 and CXCR2 (56). Research has shown that, in addition to being considerably elevated in individuals with inflammatory bowel disease, CXCL6 is also expressed more highly in those who have periodontitis (57, 58). In addition, investigations have demonstrated that ionizing radiation can significantly increase skin inflammation. Furthermore, it may promote the release of CXCL6 from fetal foreskin and adult human dermal fibroblasts, with mast cells mediating this release (59). Sun’s research found that, in the diabetic nephropathy (DN) model, by activating the JAK/STAT3 signaling pathway, CXCL6 might enhance fibrosis-related variables to hasten the development of DN renal interstitial fibrosis (60). Therefore, in treating diabetic neuropathy, CXCL6 appears to hold promise as a new therapeutic target and possible biomarker for JAK/STAT3 activation (60). In their study on PPP, Wolk et al.’s research found that IL-19 blood levels were upregulated in PPP patients and were strongly associated with the number of palmoplantar pustules (61). Activated neutrophils *in vitro* could produce IL-19 and increase CXCL6. Simultaneously, following the improvement of pharmacological therapy, PPP patients’ levels of IL-19 and CXCL6 were similarly reduced, albeit to differing degrees. It may be deduced from our research, in conjunction with earlier studies, that CXCL6 is crucial to the PPP illness process.

Initially, CCL19 was called macrophage proinflammatory human 3-β (MIP3-β). The thymus and lymph nodes are two lymphoid organs where CCL19 is strongly expressed (62). Therefore, it performs physiological and homeostatic roles in the immune system’s development (63). CCL19, which is also considered to be a shared receptor with CCL21, has a main and functional receptor called CCR7 (64). In secondary lymphoid tissues, CCR7 and CCL19 are crucial in controlling immune cell trafficking, which includes DCs and lymphocytes (65). Furthermore, CCL19 facilitates immune cell transit and compartmentalization in secondary lymphoid tissues, including T cells and DC (66). For physiological functions, CCL19 controls lymphocyte and DC homeostatic trafficking along a chemokine gradient and the recruitment of T cells expressing CCR7. Additionally, it contributes to improving antitumor immunity and T-cell responses (63). Through its anti-apoptotic activity, CCL19 not only plays a crucial role in naive T-cell homeostasis but also increases DC endocytosis capacity and suppresses the apoptosis of DC (67).On the other hand, the biological effects of CCL19/CCR7 are mediated by activating GPK/β-arrestin, which is responsible for transducing extracellular stimuli binding to intracellular signaling. As a result, CCL19/CCR7 is crucial for the immune system’s control of inflammatory bowel disorders, infection prevention, and cancer suppression (68–70). To date, not much is known about the mechanism of CCL19 in PPP illnesses, which is anticipated to be explored in future studies. A crucial immunomodulatory cytokine, interleukin-6 (IL-6), influences the etiology of several illnesses, such as cancer, autoimmune disorders, and chronic inflammatory ailments. The Kishimoto group first cloned IL-6 as B-cell stimulatory factor-2 (BSF-2) in 1986 (71, 72). IL-6 is a pleiotropic cytokine that plays an important role in many physiological and pathological processes, including immune regulation, inflammatory response, hematopoietic regulation, and tumorigenesis, and is recognized as one of the primary immunomodulatory cytokines regulating health and illness (73, 74). In addition, IL-6 is utilized clinically to assess infection control and the activity of inflammatory disorders (75, 76). Regarding immune system mechanisms, IL-6 can increase Th2 cell production and function. Th2 cell-produced cytokines, such as IL-4, IL-5, and IL-13, can also suppress Th1 cell activity, lowering the Th1/Th2 ratio and decreasing inflammation (77). Furthermore, IL-6 can strengthen the humoral immune response of the body by inducing the production of antibodies by B cells and thwarting inflammatory reactions (78, 79). Then, via controlling blood cell composition and bone marrow hematopoietic stem cell proliferation and differentiation, IL-6 can influence the inflammatory response (80, 81). For instance, IL-6 can boost the body’s ability to fight inflammation by encouraging the development of erythroid and myeloid progenitor cells, red blood cells, and white blood cells. Therefore, when it comes to therapy, the IL-6 monoclonal antibody has been used as an effective treatment for many severe patients with rheumatoid arthritis and COVID-19 (82, 83). However, it is crucial to remember that while IL-6 might benefit some situations, it can also trigger an excessive or uncontrollable inflammatory response in other conditions (77). Therefore, its role in inflammatory diseases must be judged according to the situation. In our MR study, we found that CCL19 and IL-6 played a protective role, and CXCL6 played a detrimental role in the PPP. Research on the roles of CXCL6, IL-6, and CCL19 in PPP is still lacking, and some results contradict our findings (84). These discrepancies may be attributed to the intricate interactions between inflammatory mediators and PPP, which are often regulated by various factors. Clusters of inflammatory factors rather than single cytokines may work together to cause disease. Understanding the underlying causes of the collective reaction of different inflammatory cytokine groups and how they interact with PPP will help us better reveal the intricate systems involved and develop immune-focused therapies with fewer side effects. Furthermore, researchers may find developing putative biomarkers for PPP severity or therapy helpful in response.

PPP is difficult to cure and typically takes a long time to control. Patients who are resistant to therapy and frequently relapse are discouraged from continuing. For PPP therapy, no documented recommendations or therapeutic criteria have been established as of yet. Our research indicates that CXCL6, an appealing potential biomarker and prospective therapeutic target for JAK/STAT3 signaling (60), has a negative impact on the PPP. Furthermore, tofacitinib, a JAK inhibitor, has been shown in multiple studies to alleviate refractory PPP. This treatment rapidly reduces the severity of skin lesions, such as erythema, aseptic pustules, and desquamation (85, 86). The findings of this research strongly endorse the future target of JAK/STAT3 signaling in PPP and offer genetic evidence for the use of tofacitinib in the treatment of refractory PPP.

This MR study is the first to investigate the causal relationship between systemic inflammatory cytokines and PPP using recently merged data. Our study, which provides a trustworthy pair of causal relationship links, eliminates confounders and reverses causality as much as is feasible. Moreover, our research records are obtained from the GWAS database, which is accessible to the public and contains a sizable volume of original study data, offering solid assurance for the research. Nonetheless, a few drawbacks need to be resolved in further research. First and foremost, people of European ancestry provided most of the genetic information used in this investigation. Therefore, it is possible that the results cannot be applied or generalized to other ethnic groups, and care should be taken when extending the findings to a diverse range of ethnic backgrounds. Comparable research with more racially varied cohorts is anticipated in the future. Second, given the small sample size in our validation cohort, large-scale studies are expected to verify our results even more. Third, although our Mendelian randomized method proved beneficial for determining causation, considering the inherent restrictions of MR investigations, it would be ideal for corroborating the previous findings by examining the relationship between blood levels of CCL19, CXCL6, IL-6, and the severity of PPP in a sizable cohort of PPP patients in the future. Depending on the extent of body area involvement and the severity of the disease, PPP patients may exhibit a wide range of cytokine patterns. Furthermore, combining environmental factors with genetic data in future research might be advantageous because of the complicated nature of PPP, the involvement of external variables (drugs and environment), and immunological skin problems. To accomplish this, cytokine levels in afflicted and healthy individuals must be measured and compared to known gene polymorphisms.

## 5 Conclusion

In conclusion, our bidirectional MR study suggests that there may be a causal relationship between PPP and specific circulating cytokines. Our findings would establish a solid basis for further research into pathophysiology of the cytokine that causes PPP and offer more proof in support of cytokine-targeted therapy for the illness in the future.

## Data availability statement

The original contributions presented in the study are included in the article/Supplementary material, further inquiries can be directed to the corresponding author.

## Ethics statement

The requirement of Ethical Approval was waived by the committee of the Ethics in 924th Hospital for the studies on humans because summary statistics for this article were all taken from public, readily available sources. Database permission and ethical approval were obtained. New ethics approval was not required because this study did not use any new individual-level data. The studies were conducted in accordance with the local legislation and institutional requirements. Written informed consent for participation was not required from the participants or the participants’ legal guardians/next of kin in accordance with the national legislation and institutional requirements. The human samples used in this study were acquired from gifted from another research group.

## Author contributions

CL: Writing – review & editing, Writing – original draft, Methodology, Investigation, Data curation. XingL: Writing – review & editing, Writing – original draft, Investigation, Formal analysis, Data curation. HX: Writing – review & editing, Supervision, Resources, Project administration, Funding acquisition, Formal analysis. XinL: Writing – review & editing, Writing – original draft, Supervision, Investigation, Funding acquisition.

## Footnotes

^1^ https://storage.googleapis.com/finngen-public-data-r9/summary_stats/finngen_R9_L12_PSORI_PUSTUPALM.gz
